# Synthesis and Antioxidant Activity of 2-Amino-5-methylthiazol Derivatives Containing 1,3,4-Oxadiazole-2-thiol Moiety

**DOI:** 10.1155/2013/620718

**Published:** 2013-08-19

**Authors:** Kikkeri N. Mohana, Chikkur B. Pradeep Kumar

**Affiliations:** Department of Studies in Chemistry, University of Mysore, Manasagangotri, Mysore 570006, India

## Abstract

A series of new 5-(2-amino-5-methylthiazol-4-yl)-1,3,4-oxadiazole-2-thiol derivatives **6**(**a–j**) were designed and synthesized with various substituted aldehydes. The chemical structures were confirmed by elemental analyses, FT-IR, ^1^H NMR, and mass spectral studies. The antioxidant activity of the synthesized compounds was evaluated by 2,2-diphenyl-1-picrylhydrazyl (DPPH), hydroxyl, nitric oxide, and superoxide radical scavenging assay methods. Compounds **6a**, **6e**, and **6c** showed significant radical scavenging potential due to the presence of electron donating substituent on substituted aldehydes.

## 1. Introduction

Compounds containing azomethine group (–C=N–) in the structure are known as Schiff bases which are usually synthesized by the condensation of primary amines and active carbonyl groups. Schiff bases are found to exhibit multifunctional properties, and they are able to improve various biological and pharmacological activities such as antitumor, antioxidant, and antibacterial activities [1]. Schiff bases bearing heterocyclic residues possessing excellent biological activity have attracted the attention of many researchers in recent years [2]. Due to the great flexibility and diverse structural aspects of Schiff bases, a wide range of these compounds have been synthesized and their activities have been studied [3, 4]. Many Schiff bases are known to be medicinally important and are used to design medicinal compounds [5].

1,3,4-Oxadiazoles and thiazoles are versatile leading molecules for designing potential bioactive agents. The derivatives of these molecules constitute an important family of heterocyclic compounds. Compounds bearing 1,3,4-oxadiazole nucleus are known to exhibit remarkable biological activities such as antimalarial, anticancer, anticonvulsant, and anti-inflammatory [6–12]. Thiazole nucleus is also an integral part of all the available penicillins which have revolutionized the therapy of bacterial diseases [13]. The applications of thiazoles were found in the drug development for the treatment of allergies, hypertension, inflammation, schizophrenia, bacterial, HIV infections, hypnotics, and so forth [14].

Free radicals such as superoxide, hydroxyl, and nitric oxide are the oxygen centered free radicals, and they are also called reactive oxygen species (ROS). They are generated in the human body and would cause damage to lipids, proteins, and DNA and thus may lead to various diseases such as carcinogenesis, drug-associated toxicity, and inflammation. Furthermore, radical reactions play a significant role in the development of life limiting chronic diseases such as cancer, ageing, diabetes, arteriosclerosis and others [15]. Antioxidants are molecules, natural or synthetic, capable of interacting with free radicals and stopping their chain reactions before essential vital molecules are damaged [16]. Thus, they are recently fabricated as the drug candidates to counter these multifarious diseases such as carcinogenesis, inflammation, atherogenesis, and aging in aerobic organisms [17, 18]. 

In view of these findings, it was considered of interest to undertake the synthesis of 2-amino-5-methylthiazol derivatives containing 1,3,4-oxadiazole-2-thiol moiety, hoping that these compounds might possess certain antioxidant activity. The structures of the synthesized compounds were deduced on the basis of ^1^H NMR, IR, and mass spectra. The composition of all compounds was obtained by elemental analyses. All the newly synthesized compounds were screened for their antioxidant activity by diphenylpicrylhydrazyl (DPPH), nitric oxide, hydroxyl, and superoxide radical scavenging assay methods.

## 2. Results and Discussion

### 2.1. Chemistry

**Scheme 1:**
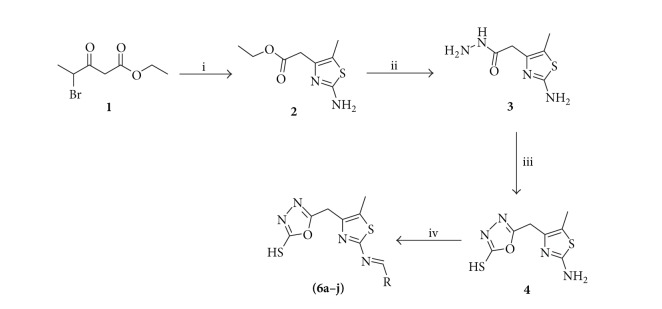
Scheme for the synthesis of new 2-amino-5-methylthiazol derivatives **6(5a–j)**. Reagents and conditions: (i) thiourea, EtOH, MW, 5 min (ii) NH_2_NH_2_ *·*2H_2_O, EtOH, reflux (iii) KOH, CS_2_, EtOH, reflux (iv) R-CHO **(5a–j)**, EtOH, reflux.

The new 2-amino-5-methylthiazol derivatives **6(a–j)** were synthesized according to Scheme 1. Reaction of ethyl 4-bromo-3-oxopentanoate **1** with thiourea in EtOH resulted in the formation of ester compound **2**. Compound **2** on further condensation with hydrazine hydrate resulted in the formation of 2-(2-amino-5-methylthiazol-4-yl) acetohydrazide **3**. Compound **3** in the presence of carbon disulfide in a basic alcohol solution yielded the desired compound **4**. The condensations of compound **4** with different aldehydes **5(a–j)** in ethanol at a reflux temperature resulted in the formation of the desired Schiff bases **6(a–j)**. TLC was run throughout the reaction to optimize the reaction for purity and completion. All the synthesized compounds were characterized using ^1^H NMR, IR, and mass spectral studies.

The ^1^H NMR spectrum of compound **2** showed a triplet signal at 1.18 ppm and quartet signal at 4.07 ppm, which supports the presence of ethyl ester group. ^1^H NMR spectrum revealed the singlet at 2.32 and 6.91 ppm corresponding to the CH_3_ and amine group, respectively. The IR spectra of compound **2** showed bands at 1684 and 3364 cm^−1^ due to C=O (ester) and NH_2_, respectively. Compound **2** on further condensation with hydrazine hydrate resulted in the formation of 2-(2-amino-5-methylthiazol-4-yl) acetohydrazide **3**. IR spectrum of compound **3** displayed absorption bands at 3316 and 3148 cm^−1^ for NH and NH_2_ groups. The ^1^H NMR spectrum of compound **3** showed signal at 9.02 ppm (s, 1H, NH) and at 6.21 and 6.86 ppm (s, 4H, 2 NH_2_). The synthesis of compound **4** involves an initial reaction between compound **3** and carbon disulfide in a basic alcohol solution followed by acidification of the reaction mixture to get the desired compound **4**. A comparative study of ^1^H NMR spectral data of compounds **3** and **4** revealed that the absence of signal at 9.02 (s, 1H, NH) and 6.21 ppm (s, 2H, NH_2_) and appearance of a signal at 13.02 ppm (s, 1H, SH) confirm the formation of product **4**. 

The reactions of compound **4** with different aldehydes **5(a–j)** were carried out in the presence of absolute ethanol to afford the compounds **6(a–j)**. The absence of NH_2_ bands in the IR spectra indicated that the synthesized compounds were obtained via condensation. The presence of C=N stretching bands at around 1603–1574 cm^−1^ corresponded to azomethine group in the synthesized compounds. ^1^H NMR spectra of all the synthesized analogues showed –CH=N– proton as singlet at 9.07–8.59 ppm, which confirmed the condensation between the amino group and aldehyde group. The signal due to methoxy (–OCH_3_) in the compound **6c** showed singlet at 3.83 ppm. The ^1^H NMR and mass spectra of the title compounds were in conformity with the assigned structure. The mass spectra of these compounds showed M+1 peaks corresponding to their molecular formula. The chemical structures and physical data of all the synthesized compounds are tabulated in Table 1.

### 2.2. Antioxidant Activity

Free radical scavenging is one of the best known mechanisms by which antioxidants inhibit lipid oxidation. DPPH, hydroxyl, nitric oxide, and superoxide radical scavenging activity evaluations are standard assays in antioxidant activity studies. The antioxidant activity of the 5-((2-amino-5-methylthiazol-4-yl) methyl)-1,3,4-oxadiazole-2-thiol derivatives **6(a–j)** was determined by these four methods using ascorbic acid (AA) and butylated hydroxyanisole (BHA) as standards.

#### 2.2.1. DPPH Radical Scavenging Activity

The *in vitro* antioxidant activity of **6(a–j)** was determined spectrophotometrically by DPPH radicals, and the results are given in Table 2. DPPH radicals are stable free radicals, and their in the presence of molecules capable of donating H atoms, their radical character is neutralized [19]. The reduction capacity of DPPH radicals was determined by the decrease in its absorbance at 517 nm, which is induced by antioxidants. On the other hand, it is well established that organic molecules incorporating an electron donating groups (amine, hydroxyl, and methoxy) can act as free radical trapping agents and are capable of opposing oxidative challenges. It can be seen from Table 2 that compounds **6a**, **6c**, **6e**, **6f**, and **6g** present the highest scavenging activity on DPPH^•^, whereas the compounds **6d**, **6h**, and **6i** exhibit moderate and **6b**, **6j**, **6k**, and **6l** showed very low scavenging activity on DPPH^•^, respectively.

All the compounds **6(a–j)** showed comparable or slight less activity to the standard (ascorbic acid). Compounds **6a** and **6e** bearing a hydroxyl group (electron donating group) at para position showed dominant DPPH activity with IC_50_ values of 14.9 and 15.0 *μ*g/mL, respectively. The presence of nitro group (electron withdrawing group) **6i** instead of a hydroxyl group in the same position exhibits less activity compared to compounds **6a** and **6e**. When methoxy groups are added in the 2, 3, or 4 positions on the phenyl ring, the antioxidant activity is increased (**6a**, **6c**, and **6e**). This activity may be correlated with the introduction of electron donor substituent which stabilizes the generated radical during oxidation.

#### 2.2.2. Hydroxyl Radical Scavenging Activity

Hydroxyl radicals are known to be the most reactive of all of the reduced forms of dioxygen and are thought to initiate cell damage *in vivo* [20]. This assay shows the ability of the compounds and standard BHA to inhibit hydroxyl radical mediated deoxyribose degradation in an *in vitro* system that consists of Fe^3+^-EDTA-ascorbate and H_2_O_2_. In this system, hydroxyl radicals generated by the Fenton's reaction attack deoxyribose and degrade into fragments that react with thiobarbituric acid (TBA) on heating to form a pink color. Hydroxyl radical (^•^OH) scavenging capacity of the compounds is directly related to its antioxidant activity. The results are shown in Table 2. The IC_50_ values of compounds **6(a–j)** in this assay were in the range of 17.9 to 43.2 *μ*g/mL. The IC_50_ values obtained are more than that of the standard. The compounds **6a** and **6e** were found to show better inhibition compared to other compounds with IC_50_ values 17.9 and 18.00 *μ*g/mL, respectively. The compounds **6h**, **6i**, and **6j** showed poor radical scavenging activity compared to the standard. The results were compared with standard BHA (15.3 *μ*g/mL) which was more effective than the compounds tested.

#### 2.2.3. Nitric Oxide Radical Scavenging Activity

In addition to reactive oxygen species, nitric oxide is also implicated in inflammation, cancer, and other pathological conditions [21]. Sodium nitroprusside (SNP) in aqueous solution at physiological pH spontaneously generates nitric oxide which interacts with oxygen to produce nitrite ions that can be estimated using Griess reagent. Scavengers of nitric oxide compete with oxygen, leading to the reduced production of nitrite ions. Suppression of the released NO may be partially attributed to direct NO scavenging. Compounds **6(a–j)** tend to decrease the amount of nitrite generated from the decomposition of SNP *in vitro.* The tested compounds showed a moderate to good nitric oxide scavenging activity. compounds **6a**, **6e** and **6c** showed the most active, but the compound 6h (IC_50_ 46.40 *µ*g/mL) showed least nitric oxide radical scavenging activity.

#### 2.2.4. Superoxide Radical Scavenging Assay

Superoxide anion radical is normally initially formed, and its effects can be magnified because it produces other kinds of free radicals and oxidizing agents [22]. The enzymatic superoxide anion radical was generated by the xanthine-xanthine oxidase reaction system. The generation of superoxide was estimated by the nitroblue tetrazolium (NBT) method. From Table 2, it is evident that all the synthesized 2-amino-5-methylthiazole derivatives **6(a–j)** were found to be moderate to weak super oxide radical scavengers. The IC_50_ values of these compounds were in the range of 17.2–48.6 *μ*g/mL. It should be noted that the activity of the compounds **6a** and **6e** was comparable to that of standard. The better activity of **6a** is due to the presence of two methoxy groups in the aromatic ring and methoxy and hydroxyl groups in **6e** of the aromatic ring. Favorable superoxide radical scavenging was found for compounds **6c**, **6f**, **6g**, and **6b** ranging from 22.2 to 28.3 *μ*g/mL, while the compounds **6h** and **6j** showed less activity. 

## 3. Conclusions

In conclusion, a series of new 2-amino-5-methylthiazol derivatives containing 1,3,4-oxadiazole-2-thiol moiety were synthesized in good yield and were characterized by different spectral studies. The synthesized compounds showed a wide range of potentially promising antioxidant activities. Compounds **6a**, **6c**, and **6e** showed significant scavenging effect against the tested free radicals.

## 4. Experimental

### 4.1. Chemistry

Melting range was determined by Veego Melting Point VMP III apparatus. Elemental analyses were recorded on VarioMICRO Superuser V1.3.2 Elementar. The FT-IR spectra were recorded using Nujal on FT-IR Jasco 4100 infrared spectrophotometer. ^1^H NMR spectra were recorded on Bruker DRX-500 spectrometer at 400 MHz using DMSO-d_6_ as solvent and TMS as an internal standard. Mass spectral data were obtained by LC/MSD Trap XCT. All solvents and reagents were purchased from Sigma-Aldrich Chemicals Pvt. Ltd.

*Synthesis of Ethyl 2-(2-Amino-5-methylthiazol-4-yl) Acetate* ***(2)***. Initially ethyl 2-(2-amino-5-methylthiazol-4-yl) acetate **(2)** was synthesized by reaction of ethyl 4-bromo-3-oxopentanoate **1** (2.23 gm, 10 mmol) with thiourea (0.76 gm, 10 mmol) in a MW test tube (20 mL) containing a magnetic stirring bar, rubber cap, and 8 mL of ethanol. The test tube was placed in the microwave cavity and subjected to MW irradiation at 50°C (100 W) for 5 min. The tube was removed and cooled to room temperature and the content was added to water (20 mL). The product was extracted into methylene chloride (30 mL) with a good yield. Yield: 74%. IR (nujol, cm^−1^): 3407 (NH_2_), 1684(C=O), ^1^H NMR (DMSO-d_6_, *δ* ppm): 6.91 (s, 2H, NH_2_), 4.07 (q, 2H, O–CH_2_–CH_3_), 3.43 (s, 2H, CO–CH_2_), 2.32 (s, 3H, CH_3_), 1.18 (t, 3H, O–CH_2_–CH_3_).

*Synthesis of 2-(2-Amino-5-methylthiazol-4-yl) Acetohydrazide* **(*3*)**. A solution of compound **2** (1.6 gm, 8 mmol) and hydrazine hydrate (0.32 mL, 10 mmol) in absolute ethanol (20 mL) was refluxed for 6 h. Upon cooling, the formed precipitate was filtered off and recrystallized from ethanol to give the compound **4**. Yield: 74%. IR (nujol, cm^−1^): 3316, 3148 (NH, NH_2_), 2352 (NH_2_). ^1^H NMR (DMSO-d_6_, *δ* ppm): 9.02 (s, 1H, NH), 6.86, 6.21 (s, 4H, 2 NH_2_), 3.17 (s, 2H, CO–CH_2_), 2.31 (s, 3H, CH_3_), 2.19 (s, 2H, NH_2_).

*Synthesis of 2-((2-Amino-5-methylthiazol-4-yl) methyl) Oxadiazole-5-thiol* ***(4)***. Compound **4** (0.93 gm, 5 mmol) was added to a solution of KOH (1.12 gm, 0.02 moL) in ethanol (30 mL), followed by the dropwise addition of carbon disulfide (CS_2_) (1.2 mL, 0.02 moL), and the yellow solution was heated and refluxed till the evolution of H_2_S ceased (18–20 h). After cooling, the solution was poured into ice-cooled water and acidified with concentrated HCl at pH 3-4. The solid was filtered, dried, and recrystallized from ethanol. Yield: 74%. IR (nujol, cm^−1^): 3368 (NH_2_), 3864, 3745, 3622, 3535, 3307, 2031, 1615, 839, 648, 662, 615. ^1^H NMR (DMSO-d_6_, *δ* ppm): 13.02 (s, 1H, SH), 7.05 (s, 1H, Ar-H), 6.45 (s, 2H, NH_2_), 3.91 (s, 2H, CH_2_), 2.31 (s, 3H, CH_3_).

*General Method for the Synthesis of Schiff Bases* ***(6a–j)***. Compound **4** (2 mmol) and the appropriate aldehyde, (2 mmol) in methanol (30 mL), and 2 to 3 mL of glacial acetic acid were refluxed for 6 h. The reaction mixture was cooled by adding ice water; the formed precipitate was filtered off, washed with water, and crystallized from ethanol to obtain the desired Schiff bases **6(a–j)**.

*5-((2-((2,4-Dimethoxybenzylidene) amino)-5-methylthiazol-4-yl) methyl)-1,3,4-oxadiazole-2-thiol* ***(6a)***. IR (nujal, cm^−1^): 1603 (–CH=N–), ^1^H NMR (DMSO-d_6_, *δ* ppm): 13.01 (s, 1H, SH), 8.61 (s, 1H, –CH=N–), 7.70–6.52 (m, 3H, Ar-H), 3.81 (s, 6H, 2 OCH_3_), 3.61 (s, 2H, CH_2_), 2.32 (s, 3H, CH_3_). MS, *m/z*: 377 (M+1). Elemental analysis found (calculated) for C_16_H_16_N_4_O_3_S_2_ (%): C, 51.05 (51.12); H, 4.28 (4.32); N, 14.88 (14.93).

*5-((2-((Benzo* *[1,3] dioxol-5-ylmethylene) amino)-5-methylthiazol-4-yl) methyl)-1,3,4 Oxadiazole-2-thiol* ***(6b)***. IR (nujal, cm^−1^): 1597. ^1^H NMR (DMSO-d_6_, *δ* ppm): 13.02 (s, 1H, SH), 8.59 (s, 1H, –CH=N–), 7.58–6.95 (m, 3H, Ar-H), 5.91 (s, 2H, O–CH_2_–O), 3.63 (s, 2H, CH_2_), 2.31 (s, 3H, CH_3_). MS, *m/z*: 361 (M+1). Elemental analysis found (calculated) for C_15_H_12_N_4_O_3_S_2_ (%): C, 49.99 (5012): H, 3.36 (3.45): N, 15.55 (15.62).

*5-((2-((4-Methoxybenzylidene) amino)-5-methylthiazol-4-yl) methyl)-1,3,4-oxadiazole-2-thiol* ***(6c)***. IR (nujal, cm^−1^): 1586 (–CH=N–). ^1^H NMR (DMSO-d_6_, *δ* ppm): 13.05 (s, 1H, SH), 8.67 (s, 1H, –CH=N–), 7.84–7.06 (m, 4H, Ar-H), 3.83 (s, 3H, OCH_3_), 3.61 (s, 2H, CH_2_), 2.30 (s, 3H, CH_3_). MS, *m/z*: 347 (M+1). Elemental analysis found (calculated) for C_15_H_14_N_4_O_2_S_2_ (%): C, 52.01 (52.13): H, 4.07 (4.18): N, 16.17 (16.21).

*5-((2-((4-Isopropylbenzylidene) amino)-5-methylthiazol-4-yl) methyl)-1,3,4-oxadiazole-2-thiol* ***(6d)***. IR (nujal, cm^−1^): 1585 (–CH=N–). ^1^H NMR (DMSO-d_6_, *δ* ppm): 13.05 (s, 1H, SH), 8.61 (s, 1H, –CH=N–), 7.75–7.35 (m, 4H, Ar-H), 3.61 (s, 2H, CH_2_), 2.87 (q, H, CH), 2.31 (s, 3H, CH_3_), 1.20 (d, 6H, CH_3_). MS, *m/z*: 359 (M+1). Elemental analysis found (calculated) for C_17_H_18_N_4_OS_2_ (%): C, 56.96 (56.98): H, 5.06 (5.16): N, 15.63 (15.74).

*4-(((4-((5-Mercapto-1,3,4-oxadiazol-2-yl) methyl)-5-methylthiazol-2-yl) imino)methyl)-2-methoxyphenol* ***(6e)***. IR (nujal, cm^−1^): 3416 (OH), 1597 (–CH=N–).^1^H NMR (DMSO-d_6_, *δ* ppm): 13.05 (s, 1H, SH), 9.13 (s, 1H, OH), 8.61 (s, 1H, –CH=N–),7.52–6.91 (s, 2H, Ar-H), 3.83 (s, 3H, OCH_3_), 3.61 (s, 2H, CH_2_), 2.30 (s, CH_3_). MS, *m/z*: 363 (M+1). Elemental analysis found (calculated) for C_15_H_14_N_4_O_3_S_2_ (%): C, 49.71 (49.79): H, 3.89 (3.96): N, 15.46 (15.56).

*2-(4-(((4-((5-Mercapto-1,3,4-oxadiazol-2-yl) methyl)-5-methylthiazol-2-yl) imino)methyl) phenyl) Ethanol* ***(6f)***. IR (nujol, cm^−1^): 3446 (OH), 1603 (–CH=N–), ^1^H NMR (DMSO-d_6_, *δ* ppm): 13.05 (s, 1H, SH), 8.62 (s, 1H, –CH=N–), 7.78–7.34 (m, 4H, Ar-H), 3.83 (t, 2H, CH_2_), 3.68 (s, 1H, OH), 3.58 (s, 2H, CH_2_), 2.77 (t, 2H, Ar-CH_2_), 2.30 (s, 3H, CH_3_). MS, *m/z*: 361 (M+1). Elemental analysis found (calculated) for C_16_H_16_N_4_O_2_S_2_ (%): C, 53.31 (15.41): H, 4.47 (4.51): N, 15.54 (15.62).

*5-((2-((4-(Dimethylamino) benzylidene)amino)-5-methylthiazol-4-yl) methyl)-1,3,4-oxadiazole-2-thiol* ***(6g)***. IR (nujal, cm^−1^): 1584 (–CH=N–). ^1^H NMR (DMSO-d_6_, *δ* ppm): 13.05 (s, 1H, SH), 8.62 (s, 1H, –CH=N–), 7.50–6.81 (m, 4H, Ar-H), 3.61 (s, 2H, CH_2_), 3.06 (s, 6H, 2 CH_3_), 2.30 (s, 3H, CH_3_). MS, *m/z*: 360 (M+1). Elemental analysis found (calculated) for C_16_H_17_N_5_OS_2_ (%): C, 53.46 (53.48): H, 4.77 (4.83): N, 19.48 (19.57).

*5-((2-((Furan-3-ylmethylene) amino)-5-methylthiazol-4-yl) methyl)-1,3,4-oxadiazole-2-thiol* ***(6h)***. IR (nujal, cm^−1^): 1597 (–CH=N–). ^1^H NMR (DMSO-d_6_, *δ* ppm): 13.05 (s, 1H, SH), 9.07 (s, 1H, –CH=N–), 7.93–6.81 (m, 3H, Ar-H), 3.60 (s, 2H, CH_2_), 2.32 (s, 3H, CH_3_). MS, *m/z*: 307 (M+1). Elemental analysis found (calculated) for C_11_H_8_N_4_O_2_S_2_ (%): C, 47.04 (47.08): H, 3.29 (3.38): N, 18.29 (18.31).

*5-((5-Methyl-2-((2-nitrobenzylidene) amino)thiazol-4-yl) methyl)-1,3,4-oxadiazole-2-thiol* ***(6i)***. IR (nujal, cm^−1^): 1578 (–CH=N–). ^1^H NMR (DMSO-d_6_, *δ* ppm): 13.05 (s, 1H, SH), 8.59 (s, 1H, –CH=N–), 8.01–7.59 (m, 3H, Ar-H), 3.61 (s, 2H, CH_2_), 2.32 (s, 3H, CH_3_). MS, *m/z*: 362 (M+1). Elemental analysis found (calculated) for C_14_H_11_N_5_O_3_S_2_ (%): C, 46.53 (46.62): H, 3.07 (3.16): N, 19.38 (19.46). 

*5-((5-Methyl-2-((pyridin-2-ylmethylene) amino)thiazol-4-yl) methyl)-1,3,4-oxadiazole-2-thiol* ***(6j)***. IR (nujal, cm^−1^): 1603 (–CH=N–). ^1^H NMR (DMSO-d_6_, *δ* ppm): 13.05 (s, 1H, SH), 8.79 (s, 1H, –CH=N–), 8.31–7.64 (m, 4H, Ar-H), 3.60 (s, 2H, CH_2_), 2.30 (s, 3H, CH_3_). *m/z*: 318 (M+1). Elemental analysis found (calculated) for C_13_H_11_N_5_OS_2_ (%): C, 49.19 (49.23): H, 3.49 (3.57): N, 22.07 (22.21).

### 4.2. Antioxidant Activity

#### 4.2.1. DPPH Method

The free radical scavenging activity of the synthesized molecules was measured in terms of hydrogen donating or radical scavenging ability using the stable radical DPPH [23]. The test samples (10–100 *μ*L) were mixed with 1.0 mL of DPPH solution and filled up with methanol to a final volume of 4 mL. Absorbance of the resulting solution was measured at 517 nm in a visible spectrophotometer. Ascorbic acid was used as the reference compound. Lower absorbance of the reaction mixture indicated higher free radical scavenging activity. Radical scavenging activity was expressed as the inhibition percentage of free radical by the sample and was calculated using the following formula:

(1) $$\begin{array}{c} \%\;\text{inhibition}=\left(\frac{\left(A_{0}-A_{t}\right)}{A_{0}}\times 100\right), \end{array}$$

where *A*_0_ is the absorbance of the control (blank, without sample) and *A*_*t*_ is the absorbance in the presence of the test samples. All tests were performed in triplicate and the results were expressed as mean values ± standard deviations.

#### 4.2.2. Hydroxyl Radical Scavenging Activity

The hydroxyl radical scavenging capacity was measured using modified method [24]. Stock solutions of EDTA (1 mM), FeCl_3_ (10 mM), ascorbic acid (1 mM), H_2_O_2_ (10 mM), and deoxyribose (10 mM) were prepared in distilled deionized water. The assay was performed by adding 0.1 mL EDTA, 0.01 mL of FeCl_3_, 0.1 mL of H_2_O_2_, 0.36 mL of deoxyribose, 1.0 mL of sample (50–250 lg/mL) each dissolved in distilled water, 0.33 mL of phosphate buffer (50 mM, pH 7.4), and 0.1 mL of ascorbic acid in sequence. The mixture was then incubated at 37°C for 1 h. About 1.0 mL portion of the incubated mixture was mixed with 1.0 mL of (10%) TCA and 1.0 mL of (0.5%) TBA containing 0.025 M NaOH and butylated hydroxyl anisole (BHA)) to develop the pink chromogen measured at 532 nm. The hydroxyl radical scavenging activity of the compounds was reported as the percentage of inhibition of deoxyribose degradation and was calculated according to the following equation:

(2) $$\begin{array}{c} \%\;\text{inhibition}=\left[\frac{\left(A_{0}-A_{t}\right)}{A_{0}}\right]\times 100, \end{array}$$

where *A*_0_  is the absorbance of the control (blank, without sample) and *A*_*t*_ is the absorbance in the presence of the sample. 

#### 4.2.3. Nitric Oxide Radical Scavenging Assay

The method of Garrat [25] was adopted to determine the nitric oxide radical scavenging activity of the synthesized molecules. Sodium nitroprusside in aqueous solution at physiological pH spontaneously generates nitric oxide which interacts with oxygen to produce nitrite ions determined by the use of Griess reagents. To 2 mL of 10 mM sodium nitroprusside dissolved in 0.5 mL phosphate buffer saline (pH 7.4) was mixed with 0.5 mL of sample solution at various concentrations (50–250 *μ*g/mL). The mixture was incubated at 25°C. After 150 min, 0.5 mL of incubation solution was withdrawn and mixed with 0.5 mL of Griess reagent [1.0 mL sulfanilic acid reagent with 1 mL of naphthylethylenediamine dichloride (0.1% w/v)]. The mixture was incubated at room temperature for 30 min. The absorbance was measured at 540 nm. The amount of nitric oxide radical was calculated using the following equation:

(3) $$\begin{array}{c} \%\;\text{inhibition of NO}=\frac{\left[A_{0}-A_{t}\right]}{A_{0}}\times 100, \end{array}$$

where *A*_0_ is the absorbance before the reaction and *A*_*t*_ is the absorbance after the reaction. 

#### 4.2.4. Superoxide Radical Scavenging Assay

Superoxide radical scavenging activity was measured as described by the reported method [26]. The assay is based on the reduction of nitroblue tetrazolium (NBT) by superoxide ions generated by the xanthine-xanthine oxidase system (X-XO). The reaction system contains 0.2 mM xanthine and 0.6 mM NBT in 0.1 M phosphate buffer of pH 7.8. The tested compounds were dissolved in methanol, and the reaction was started by the addition of XO (0.07 U mL^−1^). The extent of NBT reduction was followed spectrophotometrically by measuring the increase of absorbance at 560 nm. All experiments were replicated three times. The IC_50_ of each compound was defined as the concentration which inhibited 50% of the NBT reduction by O_2_^•−^ produced in the X-XO system.

## Figures and Tables

**Table 1:** Chemical structure and physical characterization of 2-amino-5-methylthiazol derivatives **6(a–j)**.

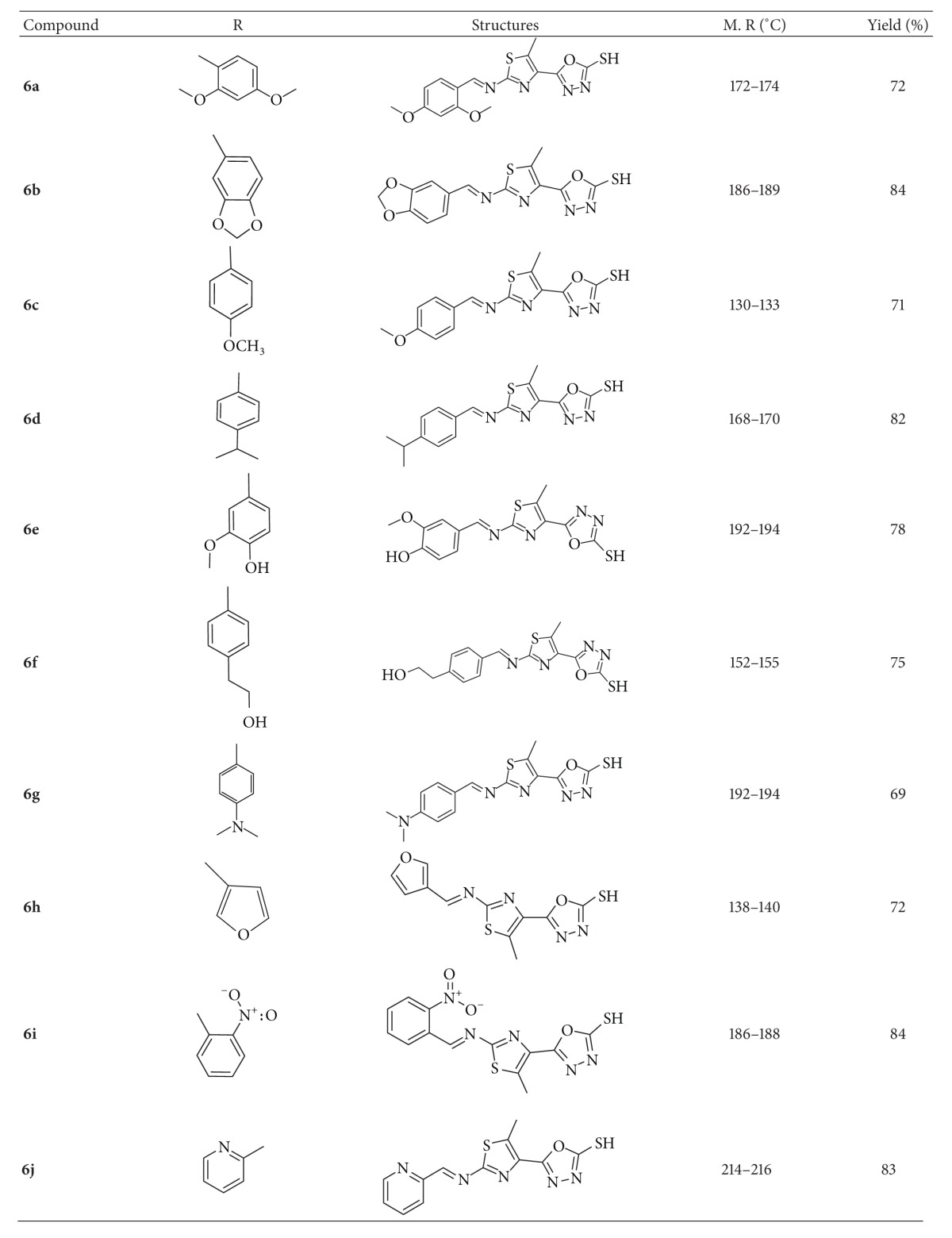

**Table 2:** IC_50_ values for evaluated antioxidant assays of examined 5-(2-amino-5-methylthiazol-4-yl)-1,3,4-oxadiazole-2-thiol derivatives **6(a–j)**.

Compounds	IC_50_ (*μ*g/mL)			
	DPPH	HO^−^	NO^−^	O_2_^−^
**6a**	14.9 ± 0.11	17.9 ± 0.20	16.4 ± 0.08	18.3 ± 0.12
**6b**	32.8 ± 0.16	35.6 ± 0.16	36.8 ± 0.41	28.3 ± 0.04
**6c**	18.4 ± 0.32	23.2 ± 0.08	20.3 ± 0.13	22.2 ± 0.34
**6d**	24.1 ± 0.08	30.1 ± 0.17	26.4 ± 0.04	32.4 ± 0.40
**6e**	15.0 ± 0.05	18.0 ± 0.65	17.9 ± 0.11	17.2 ± 0.42
**6f**	24.5 ± 0.14	30.3 ± 0.29	28.4 ± 0.20	25.2 ± 0.56
**6g**	25.7 ± 0.97	29.0 ± 0.76	28.7 ± 0.12	26.3 ± 0.25
**6h**	37.1 ± 0.26	43.2 ± 0.10	46.4 ± 0.14	46.3 ± 0.12
**6i**	31.5 ± 0.03	43.0 ± 0.03	41.3 ± 0.05	41.2 ± 0.16
**6j**	33.1 ± 0.19	40.9 ± 0.04	37.6 ± 0.13	48.6 ± 0.20
AA^a^	12.6 ± 0.43	—	—	
BHA^b^	—	15.3 ± 0.76	14.6 ± 0.11	16.1 ± 0.66

“a” refers to Ascorbic Acid and “b” refers to Butylated Hydroxyl Anisole.
